# Opening options for material transfer

**DOI:** 10.1038/nbt.4263

**Published:** 2018-10-01

**Authors:** Linda Kahl, Jennifer Molloy, Nicola Patron, Colette Matthewman, Jim Haseloff, David Grewal, Richard Johnson, Drew Endy

**Affiliations:** 1 Linda Kahl, David Grewal, Richard Johnson, and Drew Endy are at the BioBricks Foundation, San Francisco, California, USA., ,; 2 Jennifer Molloy and Jim Haseloff are at the Department of Plant Sciences, University of Cambridge, Cambridge, UK., ,; 3 Nicola Patron is at the Earlham Institute, Norwich, UK., ,; 4 Colette Matthewman is at the John Innes Centre, Norwich, UK., ,; 5 David Grewal is also at Yale Law School, New Haven, Connecticut, USA., ,; 6 Richard Johnson is also at Global Helix, Washington, DC, USA., ,; 7 Drew Endy is also at the Department of Bioengineering, Stanford University, Stanford, California, USA., ,

**Keywords:** Intellectual-property rights, Business

## Abstract

The Open Material Transfer Agreement is a material-transfer agreement that enables broader sharing and use of biological materials by biotechnology practitioners working within the practical realities of technology transfer.

**Supplementary information:**

The online version of this article (doi:10.1038/nbt.4263) contains supplementary material, which is available to authorized users.

Material-transfer agreements (MTAs) underlie the legal frameworks within which biotechnology practitioners define the terms and conditions for sharing biomaterials ranging, for example, from plasmid DNA to patient samples. If MTAs are easy to use and well adapted to the needs of individual researchers, institutions, and broader communities, then more sharing, innovation, and translation can occur. However, the MTA frameworks currently in place were developed in the 1990s—before widespread adoption of the World Wide Web, genome sequencing, and gene synthesis—and are not always well adapted for contemporary research and translation practices or aligned with social objectives.

Here, we introduce a new MTA, the Open Material Transfer Agreement (OpenMTA), that relaxes restrictions on the redistribution and commercial use of biomaterials while maintaining aspects of standard MTAs that support widespread adoption (for example, incorporation into semiautomated administration systems). In developing the OpenMTA, our motivation was to realize a simple, standardized legal tool for sharing biological materials as broadly as possible without undue restrictions, while respecting the rights of creators and promoting safe practices and responsible research. Importantly, we wanted the tool to work within the practical realities of technology transfer and to be sufficiently flexible to accommodate the needs of many groups globally (for example, providing support for international transfers and compatibility with public and philanthropic funding policies).

**Traditional MTAs**

Currently, the most used MTA in biology and biotechnology is the Uniform Biological Material Transfer Agreement (UBMTA), which was developed and widely adopted in the 1990s (https://www.ott.nih.gov/resources/). The UBMTA represented a major step forward in providing a standard template intended to help decrease administrative transaction costs for material exchange among academic research institutions. Despite widespread adoption of the UBMTA, many institutions continue to insist on MTAs specific to their own institution^1^. In many cases, the effort required to implement MTAs has increased, because the complexity of included provisions requires protracted negotiation. Such transaction costs not only impede the distribution of materials within and beyond research communities but also place an unnecessary burden on technology transfer offices, thus leading some institutions to adopt a 'no MTA' policy^2,3^. Although eliminating the explicit execution of MTAs decreases the apparent administrative burden, the resulting reality is less than ideal, because MTAs also help with provenance tracking, recognition, and quality control. In addition, because some no-MTA policies state that transfers made without an explicit agreement default to the terms of the UBMTA, and because researchers may share materials informally without knowing that such terms apply by default^4^, the ability of everyone to legitimately use and further develop materials remains in limbo, at best.

Most widely used MTAs place two restrictions on material transfers, neither of which is often useful or desired (Table 1). First, MTAs typically disallow redistribution of materials (i.e., so-received materials cannot formally be shared with others). Second, any and all commercial uses of the so-received biomaterials are specifically prohibited. Although these two restrictions are appropriate for materials that require tight control of provenance for reasons of safety, security, or commercialization, such restrictions make little sense for most of the materials used widely throughout research (for example, basic samples, strains, or plasmids). Because the potential commercial value of most widely used materials is quite low, and MTAs are unlikely to ever be monitored and enforced^1^, blanket restrictions on redistribution and commercial use create unnecessary barriers and costs within research communities and to society at large.

**Table 1:** Comparison of features and terms for standard MTAs^a^

Features and terms of transfer	UBMTA (1995)	SLA (1999)	Science Commons (2005–2009)	OpenMTA (2018)
**Similarities**				
Use for research and teaching	Yes	Yes	Yes	Yes
Attribution	Yes	Yes	Yes	Yes
Compliance with laws and regulations	Yes	Yes	Yes	Yes
No warranty (for example, third-party rights)	Yes	Yes	Yes	Yes
Recipient assumes liability	Yes	Yes	Yes	Yes
Recipient indemnifies provider	No	No	No	No
Reach-through rights or restrictions	No	No	No	No
Fees for preparation and distribution (optional)	Yes	Yes	Yes	Yes
Fees for royalties	No	No	No	No
Provenance tracking	Yes	Yes	Yes	Yes
Alignment with policies of public and private funders of research	Yes	Yes	Yes	Yes
**Differences**				
Academia or nonprofit only	Yes	No	No	No
Ease of use internationally	No	Yes	Yes	Yes
Distribution of materials or derivatives	No	No	No	Yes
Use for commercial purposes	No	No	No	Yes

^a^UBMTA, Uniform Biological Material Transfer Agreement (https://www.ott.nih.gov/resources/); SLA, NIH Simple Letter Agreement (https://www.ott.nih.gov/resources/); Science Commons MTA^19^.

For example, with the ongoing emergence of a second generation of biotechnology practitioners who are increasingly empowered by information-sharing networks and DNA sequencing and synthesis capacities—which together make genetic information and material interconvertible—both the 'no redistribution' and 'no commercial use' restrictions can be quite harmful. As one example, over the past 15 years, the international genetically engineered machines competition (http://igem.org/) has developed into a genetic engineering 'olympics' involving ∼6,000 students per year, in which students freely share biological materials under a get-and-give policy. Many of these students, having just prototyped a compelling biotechnology application, then wish to start a company to translate their innovation. Yet, the materials that they themselves developed via an open and collaborative community cannot be directly used in their subsequent commercial activities.

As a second specific example, Addgene has become a major public-benefit plasmid-sharing resource; it has shipped more than 950,000 plasmids to over 6,400 institutions in 93 countries since starting in 2004. Technology-transfer offices are enormously appreciative of the services that Addgene provides, which include electronic processing of MTAs. Yet, because the UBMTA was the default standard at the time Addgene's electronic MTA system was developed, commercial biotechnology researchers are unable to directly access most of the resources available via Addgene. This MTA-induced limitation arises even though many of the Addgene-hosted materials are not subject to any specific proprietary interest. Although well-funded commercial groups can gain access to such materials via *ab initio* DNA synthesis starting from sequences available on Addgene's website or other public databases, the companies that might benefit the most from such materials—the small start-up or midsized companies representing the lifeblood and future of biotechnology—are left out. Defaulting to restrictive terms that may not be uniformly necessary or appropriate is common with other repositories, including nonprofit culture collections, biobanks, gene banks, seed banks, and other plasmid repositories that have been established in recognition of their importance to various biotechnology communities.

Making research tools broadly available for use without restriction can accelerate the pace of research, improve the reproducibility of results, and save the time and costs that would otherwise be incurred by researchers recreating tools and materials in their own laboratories. Today, researchers in many branches of biotechnology including synthetic biology, genomics, and regenerative medicine are creating nucleic acid–based tools as well as other tools intended for widespread use and contribution to the public domain. Examples include molecular probes for drug discovery from the Structural Genomics Consortium, pluripotent stem cell lines from Boston University's Center for Regenerative Medicine, materials from the Montreal Neurological Institute and Hospital, functional genetic elements from the BIOFAB project, DNA part collections from OpenPlant, and the bionet.io and Free Genes projects from the BioBricks Foundation^5,6,7,8,9,10^. The desire of these researchers and organizations to share materials freely is rooted in the idea that, although there may be limited value in each of these tools individually, there is great value in their widespread use by researchers and others collectively. This idea is particularly relevant to synthetic biology, in which standards have been developed for the interoperability of modular DNA components or parts, and in which remixing of numerous parts is normal practice^11,12^. Nonetheless, accessing materials remains difficult and time consuming or impossible, thus leading to delays or lost opportunities.

**Drafting the OpenMTA**

The OpenMTA was developed as a collaborative effort led by the BioBricks Foundation and the OpenPlant Synthetic Biology Research Centre. We began by convening a working group comprising researchers, technology-transfer professionals, social scientists, legal experts, and others interested in creating a legal framework that could improve sharing of biomaterials. This group, which met in person in June 2015 and continued with scheduled online meetings, developed the design goals for the OpenMTA, including access, attribution, reuse, redistribution, and nondiscrimination (Fig. 1). These five design goals were selected to be consistent with the Open Definition, which aims to “make precise the meaning of 'open' with respect to knowledge, promoting a robust commons in which anyone may participate, and interoperability is maximized” (https://opendefinition.org/). Additional design goals included issues of safety and, in particular, the sharing of biomaterials in an international context. For example, we wanted the agreement to be usable by institutions worldwide (i.e., to not be US- or UK-centric). After the design goals were in place, we drafted the legal text for the OpenMTA.

**Figure 1:**
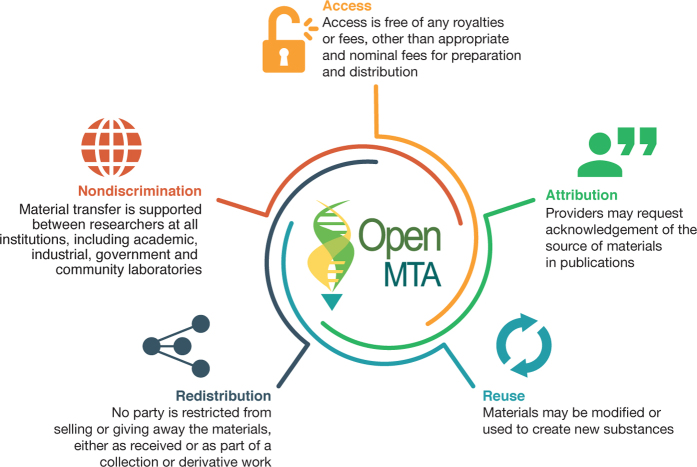
Design goals for the OpenMTA. The design goals for the OpenMTA reflect the principles of 'openness' set out in the Open Definition (https://opendefinition.org/). These design goals, together with additional goals to ensure safety and enable sharing of biomaterials in an international context, were used to help guide the drafting of the legal text for the OpenMTA.

We used the text of the UBMTA as a starting point, removing any aspects that did not meet the design goals, and adding or refining text to strengthen aspects as needed. The resulting OpenMTA drafts went through several rounds of revision, beginning with review by the offices responsible for transfer of materials from OpenPlant, including Cambridge University, Cambridge Enterprise, and the John Innes Centre. Additional input was solicited by e-mail and by contacting the members of Praxis Unico and the Association for University Technology Managers. Revisions continued until the received comments reflected no new issues, at which point the OpenMTA text was officially posted online with digital-signature capability (http://openmta.org).

The OpenMTA has many features in common with other standard MTA templates, such as provisions on use, compliance, and liability, that help protect the provider and clarify the responsibility of the recipient (Table 1). Like all MTAs, the OpenMTA is a contract—a bargained-for exchange of consideration—wherein materials from the provider are given in exchange for a promise by the recipient to abide by the terms of the agreement, including attribution, reporting back, and payment of a fee for processing if requested. Although litigation of MTAs is exceedingly rare, a framework based on providing material in exchange for a promise meets the legal standards for adequate consideration^1^. As part of the promise, the OpenMTA includes a provision that requires recipients of the materials to “ensure compliance with all applicable laws, rules, and regulations.” This provision was included to provide flexibility and stability in use of the OpenMTA because, as is the case for all MTAs, the transfer of specific materials may be subject to laws, rules, and regulations that are context dependent, jurisdictional, and subject to change over time (for example, access and benefit-sharing obligations arising from the Convention for Biodiversity and Nagoya Protocol (https://www.cbd.int/abs/)). The OpenMTA also includes an optional 'catch-all' term specifying “information relevant to the status of the Material is provided in an attachment.” This optional term, suggested during the iterative review process as a means to capture unique or unforeseen circumstances, could be used to notify the recipient of any additional obligations that might apply to use of the material (for example, uses specified in patent claims). Finally, because the OpenMTA includes the same 'no warranty' provision as that provided in the UBMTA and other standard agreements, the recipients remain responsible for conducting their own due diligence for their use of the materials in their jurisdiction.

Differences between the OpenMTA and other standard templates arise via differences in design—specifically that researchers be allowed to use the materials for any lawful purpose, including commercial purposes, and may also redistribute the materials to others, subject to reporting back if requested by the providing institution. Reporting back was included as an optional term because technology transfer offices expressed different preferences. Some wanted reporting back as a means to measure the influence of research materials made freely available to others, whereas others did not want to track the materials beyond the first transfer.

Although the OpenMTA can be used as an integrated agreement, the online version of the OpenMTA is structured as a Master Agreement that can be approved at an institutional level. The online Master Agreement ensures consistency in the use of the OpenMTA (i.e., no altering of terms or editing), and also provides transparency for the individuals and institutions that have become signatories (i.e., signatories can be listed online for easy reference). After an institution becomes a signatory to the OpenMTA Master Agreement, transfers can be made with the use of an implementing letter. Doing so simplifies the material-transfer process by eliminating the need for review of terms and provides the documentation necessary for provenance tracking.

Importantly, we note that becoming a signatory to the OpenMTA Master Agreement provides an institution and its researchers the option of transferring materials under the terms of the OpenMTA but does not obligate its exclusive use. Institutions retain full discretion to handle the transfer of specific materials on a customized basis. Institutions also retain the discretion to designate authorized signatories for the implementing letter. In other words, use of the OpenMTA is not mandatory, even for signatory institutions, and institutional signatory authority is still required unless the institution decides otherwise. Additionally, because the OpenMTA does not include a 'viral' clause, institutions may accept incoming materials under the OpenMTA, use or modify so-received materials, and then subsequently redistribute the materials or derivatives under the same or different terms. This additional flexibility supports broad use of materials made available under the OpenMTA, even in cases in which more restrictive terms are best suited for redistribution.

As a standard template, the OpenMTA lends itself to automation that can further accelerate and simplify MTA processing while providing a less restrictive option for material transfer as appropriate. Within centralized repositories such as Addgene, for example, the OpenMTA should be able to be incorporated as an option alongside the UBMTA so that researchers and institutions can easily select the terms best suited for their needs. Additionally, the provenance-tracking capability inherent in Addgene's electronic MTA system^13,14^, with the necessary permissions, could provide increased transparency in the dissemination of research tools and help inform science policy. Stated differently, institutions may choose to use a centralized repository to distribute materials under the OpenMTA to be readily recognized for reaching the widest possible audience and enabling the greatest social good.

Incorporation of the OpenMTA within other electronic platforms, such as the MTAShare platform developed at Vanderbilt University (https://cttc.co/inventors/mtashare/) and the Transfer Agreement Dashboard hosted by the US National Institutes of Health^15^, could also enable less restrictive options for sharing biomaterials as appropriate. These platforms are designed for direct transfer of materials from one institution to another and provide an avenue for researcher-led sharing of materials. Although researcher-led sharing of materials may lack the quality control of centralized repositories, such an approach is practically essential for materials undergoing rapid iterative changes or to support broad collaborations and rapid scaling. Technology-transfer offices could still review and approve such transfers, and paperwork and individual negotiations could be replaced by electric communications and selection from a set of standard MTA templates. Such electronic platforms could also offer provenance tracking, so that researchers and their institutions would be better able to make informed choices about the materials they use in their research.

**Use of the OpenMTA**

We anticipate that the OpenMTA will be most useful for the transfer of biomaterials used in precompetitive research, such as plasmids, strains, and samples, whose quantities are not limited, owing to the easily replicated nature of the materials, and for which the value of individual materials is relatively low, owing to alternative sourcing options. We further anticipate that the OpenMTA may be well suited as a default institutional policy in contexts in which most materials are intended to be freely shared. We note that the OpenMTA is not suitable for all transfers, such as materials that are in limited quantity or subject to strict biosecurity regulations.

The OpenMTA does allow for recovery of costs associated with preparation and distribution of materials and therefore could also be used for the transfer of research reagents such as antibodies, cell lines, and fluorescent proteins for which patents have expired or were never sought. Indeed, the introduction of OpenMTA for sharing biological materials is particularly timely in that many patented materials are now entering the public domain as patents expire. As one example, a collection of patents on green fluorescent proteins initially aggregated and outlicensed by GE Healthcare Lifesciences^16^ have all expired (Table 2). Moreover, patent claims to nucleic acid sequences are now subject to heightened scrutiny and, if issued at all, are drawn much more narrowly than in the past^17,18^.

**Table 2:** Patents on GFP initially aggregated and outlicensed by GE Healthcare

Patent No.	Title	Legal status	Expiration date
US 5,491,084	Uses of green fluorescent protein	Expired	2013-09-10
US 6,146,826	Green fluorescent protein	Expired	2017-11-14
US 5,625,048 US 5,777,079 US 6,319,669 US 6,066,476	Modified green fluorescent proteins Modified green fluorescent proteins Modified green fluorescent proteins Modified green fluorescent proteins	Expired Expired Expired Expired	2014-11-10 2014-11-10 2014-11-10 2014-11-10
US 5,804,387 US 6,090,919	FACS-optimized mutants of the green fluorescent protein FACS-optimized mutants of the green fluorescent protein	Expired Expired	2016-02-01 2017-01-31
US 5,958,713	Method of detecting biologically active substances by using GFP	Expired	2015-01-31
US 5,968,738	Two-reporter FACS analysis of mammalian cells using GFP	Expired	2015-12-06
US 5,994,077	Fluorescence-based isolation of differentially induced genes	Expired	2015-01-31
US 6,054,321 US 6,077,707 US 6,124,128 US 6,403,374	Long wavelength engineered fluorescent proteins Long wavelength engineered fluorescent proteins Long wavelength engineered fluorescent proteins Long wavelength engineered fluorescent proteins	Expired Expired Expired Expired	2016-08-16 2016-08-16 2016-08-16 2016-08-16
US 6,172,188 US 6,818,443 US 7,314,915	Novel fluorescent proteins Novel fluorescent proteins Novel fluorescent proteins	Expired Expired Expired	2015-09-22 2015-09-22 2015-09-22
CA 2232727	Novel variants of green fluorescent protein, GFP	Expired	2016-01-31
EP 0804457 EP 0815257 EP 0851874	Modified green fluorescent proteins A method of detecting biologically active substances Novel variants of green fluorescent protein, GFP	Expired Expired Expired	2014-11-10 2016-01-31 2016-01-31
JP 3283523 JP 3535177	Modified green fluorescent protein A new variant of a GFP is a green fluorescent protein	Expired Expired	2015-11-13 2016-01-31

We have received numerous inquiries about the suitability of the OpenMTA for the transfer of human-derived materials. Additional complexities including privacy, consent, and institutional-review-board approval must be addressed, and we are working with others to develop an OpenMTA for such materials. Extending these efforts to enable more open sharing of induced pluripotent stem cell lines, for example, in coordination with national and international registries, could vastly accelerate the development of useful biomedical applications.

Practically, the OpenMTA is already being used via the BioBricks Foundation's Free Genes project, wherein sequences requested by the synthetic biology research community are synthesized and made available without cost. The OpenPlant Synthetic Biology Research Centre plans to use the OpenMTA to distribute vectors that incorporate a common syntax with wide acceptance in the area of plant biotechnology. The online OpenMTA Master Agreement is already gaining traction, and it includes initial signatories from academic research institutions, companies, and community labs. We invite more signatories and welcome public comments on the OpenMTA Master Agreement, which is freely available via http://openmta.org and as Supplementary Data. Comments may be addressed to The OpenMTA Project, BioBricks Foundation, 77 Van Ness Avenue, Ste. 101-1626, San Francisco, California, 94102, USA, or sent by email to openmta@biobricks.org or to either corresponding author.

## Supplementary information

Supplementary Data Supplementary Data (PDF 130 kb)
