# Supplementary material for: Opening options for material transfer
Source: Nat Biotechnol. 2018 Oct 1;36(10):923–7. doi: 10.1038/nbt.4263 (PMC6871013; doi:10.1038/nbt.4263)
Supplement: Supplementary Data — (PDF 130 kb) [file 41587_2018_BFnbt4263_MOESM3_ESM.pdf]

Upon execution of an Implementing Letter in the form attached, which specifies the materials to be transferred, this organization agrees to be bound by the terms of the attached Open Material Transfer Agreement ("OpenMTA") published at https://biobricks.org/open-material-transfer-agreement/ on March 15, 2018.

Attachments:

Open Material Transfer Agreement (OpenMTA)

OpenMTA Implementing Letter

Organization:

Address:

Phone:

Email:

Name of Authorized Official:

Title of Authorized Official:

Signature of Authorized Official:

Date:

Please return an executed copy of this Master Agreement to: The OpenMTA Project, BioBricks Foundation, 77 Van Ness Avenue, Ste. 101-1626, San Francisco, California, USA, 94102. The BioBricks Foundation will be maintaining signed originals and the official list of signatory organizations.

Nature Biotechnology: doi:10.1038/nbt.4263

Open Materials Transfer Agreement (OpenMTA)

For the Transfer of Biological Materials and Associated Data

This Open Material Transfer Agreement (the "OpenMTA") and the attached Implementing Letter (the "Implementing Letter" and, together with the OpenMTA, the "Agreement") is entered into between the Provider and the Recipient (or the "Parties", as further identified in the Implementing Letter) and governs the exchange and use of the certain materials specified in this Agreement between the Parties. The provisions of this OpenMTA shall be given precedence in interpretation in the event of any conflict between this OpenMTA and the Implementing Letter.

I. DEFINITIONS:

1. **Provider**: Organization providing the Original Material. The name and address of this party will be specified in an implementing letter.

2. **Provider Scientist**: The name and address of this party will be specified in an implementing letter.

3. **Recipient**: Organization receiving the Original Material. The name and address of this party will be specified in an implementing letter.

4. **Recipient Scientist**: The name and address of this party will be specified in an implementing letter.

5. **Original Material**: The description of the Material being transferred will be specified in an implementing letter.

6. **Material**: Original Material, Progeny, and Unmodified Derivatives. The Material shall not include: (a) Modifications, or (b) other substances created by the Recipient through the use of the Material, which are not Modifications, Progeny, or Unmodified Derivatives.

7. **Progeny**: Unmodified descendant from the Material, such as virus from virus, cell from cell, or organism from organism.

8. **Unmodified Derivatives**: Substances created by the Recipient which constitute an unmodified functional subunit or product expressed by the Original Material. Some examples include: subclones of unmodified cell lines, purified or fractionated subsets of the Original Material, proteins expressed by DNA/RNA supplied by Provider, or monoclonal antibodies secreted by a hybridoma cell line.

9. **Modifications**: Substances created by the Recipient which contain/incorporate the Material.

10. **Commercial Purposes**: The sale, lease, license, or other transfer of the Materials or Modifications to a for-profit organization. Commercial Purposes shall also include uses of the Material or Modifications by any organization, including Recipient, to perform contract research, to produce or manufacture products for general sale, or to conduct research activities that result in any sale, lease, license, or transfer of the Material or Modifications to a for-profit organization.

Nature Biotechnology: doi:10.1038/nbt.4263

II. TERMS AND CONDITIONS OF THIS AGREEMENT:

The Provider is making the Material available as a service to the research community. The Recipient may use the Material for any lawful purpose, including Commercial Purposes, in accordance with the following terms and conditions:

1. **Use**: The Recipient shall use, store, and dispose of the Material and any Modifications in accordance with good laboratory practice and shall ensure compliance with all applicable laws, rules, and regulations, including laws, rules, and regulations governing export control and safety.

2. **Attribution**: The Recipient agrees to provide appropriate acknowledgement of the source of the Material as requested by the Provider. Any request for attribution will be specified in an implementing letter.

3. **Distribution**: The Recipient may distribute the Material and substances created by the Recipient through use of the Material, including Progeny, Unmodified Derivatives, and Modifications, without requesting consent from the Provider. Recipient agrees to notify the Provider of any distributions of the Material to third parties as requested by the Provider. Any request for notification will be specified in an implementing letter.

4. **Fees**: The Material is provided at no cost, or with an optional transmittal fee solely to reimburse the Provider for its preparation and distribution costs. If a fee is requested, the amount will be specified in an implementing letter.

5. **No Implied License**: Except for the rights expressly granted herein, the Recipient agrees that no other rights or licenses, whether express or implied, are granted to the Recipient under any patent, patent application, or other proprietary right of the Provider. As between the parties, each retains all right, title, and interest in works and inventions made by its personnel, and nothing herein shall be construed to transfer ownership of any invention, patent, patent application, or other proprietary right.

6. **Liability**: Except to the extent prohibited by law, the Recipient assumes all liability for damages that may arise from the use, storage or disposal of the Material. The Provider will not be liable to the Recipient for any loss, claim or demand made by the Recipient, or made against the Recipient by any other party, due to or arising from the use of the Material by the Recipient, except to the extent permitted by law when caused by the gross negligence or willful misconduct of the Provider.

7. **No Warranties**: Any Material delivered pursuant to the Agreement is understood to be experimental in nature and may have hazardous properties. THE PROVIDER MAKES NO REPRESENTATIONS AND EXTENDS NO WARRANTIES OF ANY KIND, EITHER EXPRESSED OR IMPLIED. THERE ARE NO EXPRESS OR IMPLIED WARRANTIES OF MERCHANTABILITY OR FITNESS FOR A PARTICULAR PURPOSE, OR THAT THE USE OF THE MATERIAL WILL NOT INFRINGE ANY PATENT, COPYRIGHT, TRADEMARK, OR OTHER PROPRIETARY RIGHTS.

Nature Biotechnology: doi:10.1038/nbt.4263

Template of OpenMTA Implementing Letter

The purpose of this letter is to provide a record of the material transfer, to memorialize the agreement between the Provider Scientist (identified below) and the Recipient Scientist (identified below) to abide by all terms and conditions of the Open Material Transfer Agreement ("OpenMTA"), and to certify that their respective organizations have accepted and signed an unmodified copy of the OpenMTA. The Authorized Officials of the Provider and Recipient organizations must sign this letter if the Provider Scientist or Recipient Scientist are not authorized to certify on behalf of their respective organizations.

The Recipient Authorized Signatory should sign two copies of this letter and forward to the Provider. The Provider Authorized Signatory should sign both copies of the letter and return one signed copy to the Recipient. The Provider Scientist will then forward the material to the Recipient Scientist.

**Provider** (organization providing Original Material)	**Recipient** (organization receiving Original Material)
Name:<br>Address:	Name:<br>Address:

**Provider Scientist**	**Recipient Scientist**
Name:<br>Title:	Name:<br>Title:

**Additional Information**	**Shipping Address**
	Name:<br>Address:

**Original Material (**description of the material being transferred, including any identifiers)

If checked, the following optional terms apply to this Agreement:

☐ A transmittal fee of __________________ shall be paid by Recipient to Provider, for preparation and distribution costs.

☐ Recipient agrees to provide appropriate acknowledgement of the source of the Material in all publications.

☐ Recipient agrees to notify the Provider upon redistribution of the Material to third parties.

☐ Information relevant to the status of the Material is provided in an attachment.

Nature Biotechnology: doi:10.1038/nbt.4263

This Implementing Letter is effective when signed by all parties. The parties executing this Implementing Letter certify that their respective organizations have accepted and signed an unmodified copy of the OpenMTA, and further agree to be bound by its terms, for the transfer specified above.

Provider Authorized Signatory

I hereby certify that the Provider organization has accepted and signed an unmodified copy of the OpenMTA, and further agree to be bound by its terms, for the transfer specified above (May be the Provider Scientist if authorized by the Provider organization):

Signature

Print Name

Title

Date

Recipient Authorized Signatory

I hereby certify that the Recipient organization has accepted and signed an unmodified copy of the OpenMTA, and further agree to be bound by its terms, for the transfer specified above (May be the Recipient Scientist if authorized by the Recipient organization):

Signature

Print Name

Title

Date

Nature Biotechnology: doi:10.1038/nbt.4263
